# Spatial distribution, regional differences, and dynamic evolution of the medical and health services supply in China

**DOI:** 10.3389/fpubh.2022.1020402

**Published:** 2022-09-23

**Authors:** Baoqi Chen, Fulei Jin

**Affiliations:** School of Economics, Shandong University of Finance and Economics, Jinan, China

**Keywords:** medical and health services, supply level, regional equity, evolution trend, health policy

## Abstract

The imbalance of medical and health services supply (MHSS) is a significant public health concern as regional economic development disparities widen in China. Based on the provincial panel data of medical and health services, this paper constructed an evaluation index system and used the two-stage nested entropy method to measure the MHSS level of 31 provinces in China from 2005 to 2020. Then we used the standard deviation ellipse, Dagum Gini coefficient, β convergence model, kernel density estimation and Markov chain to investigate the spatial distribution, regional differences, and dynamic evolution of MHSS. According to the results of these analysis, the conclusions are drawn as follows: (1) In general, the MHSS level in China showed a significant up-ward trend from 2005 to 2020. However, the MHSS level among different provinces showed a non-equilibrium characteristic. (2) Regional comparison shows that the eastern region had the highest level, and the central region had the lowest level. The eastern and central regions presented polarization, while the western region showed unremarkable gradient effect. (3) During the period, the overall regional differences, intra-regional differences, and inter-regional differences of MHSS level all showed convergence. (4) The economic development, urbanization rate, fiscal self-sufficiency rate, and foreign direct investment had significant impacts on the convergence. (5) The provinces with high levels had the positive spillover effect. The findings of this paper provide theoretical supports for optimizing the allocation of health resources and improving the equity of MHSS.

## Introduction

Health is a basic human right and a basic condition for human survival and social development. Providing fair medical and health services to ensure the health of the whole people is one of the unshirkable responsibilities and important functions for governments of all countries. Since the reform and opening-up, China has made remarkable achievements in economic and social development, but the problem of imbalanced development in the field of basic public services has become increasingly prominent, including the medical and health services supply (MHSS). For example, in 2020, the number of licensed or assistant doctors per 10,000 people in Beijing was 49, while there was only 25 in Yunnan in the same period. The imbalanced distribution of health resources is closely related to the safety and health of the people. It not only violates the principle of social fairness and justice, but also deviates from the Chinese government's vision of national health. To solve the long-term imbalanced and insufficient supply of health resources, in recent years, China' central government has reformed the medical and health services system and successively implemented a series of strategies such as “Equalization of Basic Public Services”, “Balanced Regional Development” and “Healthy China 2030”. Against this backdrop, research on the spatial-temporal characteristics of the MHSS in China is of great significance for regional equity. Therefore, a series of quantitative analysis tools are used to investigate the spatial distribution, regional differences, and dynamic evolution of the MHSS level in China. This paper not only provides a clear outline for decision makers to learn the characteristics associated with regional MHSS in China but can also aid in the evaluation of existing health policies to provide a practical reference for balancing the allocation of health resources among regions.

Since the 1970s, the fairness of MHSS has gradually attracted scholars' attention. Townsend (1) first raised the issue of unfairness in the field of MHSS and confirmed its existence. Subsequently, many scholars conducted their research. For instance, Smith (2) used the data of health care services in the United States to study whether there was unfairness in the types of health care services provided between poor and affluent areas. The study found that the health sector tended to provide chronic disease screening and family doctor services in poor areas, but provides more rehabilitation and mental health services in affluent areas. Rosero (3) used a traditional access measurement method based on the distance from the nearest facility and proposed a more comprehensive access index. The study found that the accessibility and fairness of outpatient care in Costa Rica had been greatly improved from 1994 to 2000, in which the reform of health system played a positive role. In general, after nearly half a century of development, the research on the fairness of medical and health services has made great progress. These studies covered country level, community level, and city level (4–6), including various fields such as the balance of medical resource allocation, the fairness of medical services, and the accessibility of medical facilities (7–9), and the research objects involved developed or developing countries such as Canada, the United States, Spain, Italy, Argentina, India, etc. (10–13).

In China, the problem of imbalanced allocation of health resources has gradually emerged, which has attracted the attention of scholars. Zheng (14) analyzed the regional differences of basic medical and health resources in China, and found that the regional differences of the allocation level of basic medical and health resources in China are large, but the differences had shown a trend of decreasing fluctuations since 2008. Xin (15) constructed a comprehensive evaluation system to measure the MHSS level in China, and also found that regional differences are gradually narrowing. Huang (16) used the Gini coefficient method to evaluate the fairness of maternal and child health resource allocation in Hunan Province. It was found that maternal and child health resource allocation is fair in the demographic dimension with the Gini values all < 0.3, but unfair in the geographical dimension. It can be found that although some studies focus on a certain local area or a certain aspect of medical and health services (16–20), and some studies focus on the whole medical and health services throughout the country (14, 15), all show that the imbalance of distribution among different regions is one of the main causes of the inequality (21, 22).

In addition, as China's urban-rural dual development structure leads to huge development differences, some scholars focus on the urban-rural dimension. Due to the impact of the dual public services supply mechanism, the distribution of health resources between urban and rural areas is imbalanced, and there are differences in the accessibility of health services between urban and rural residents (23–25). This imbalance is reflected in many aspects, such as financial health expenditure, human resources, and material resources (26–28). Therefore, the gap between urban and rural areas should be taken into account when evaluating the medical and health services supply in China. Some other scholars have paid attention to the accessibility difference of services in different regions of the same city. For instance, taking Beijing as a case study, Lu (29) found that China's referral reform in the hierarchical medical system has improved the overall accessibility of public hospitals in Beijing, but aggravated the inequality of access to medical resources between towns and villages at the same time.

In terms of the evaluation of MHSS, due to the one-sided nature of a single category of indicators, constructing a comprehensive index evaluation system has become the mainstream evaluation way in the field of medical and health services (30). Compared with the method that only select a single category of indicators, it is more reliable and closer to the objective reality (31), and widely used in the evaluation of medical and health services. For instance, Wang (11) used the cluster analysis method to form a comprehensive evaluation system for nine parts including per capita health care expenditure, and studied the convergence of health care expenditure in the United States. The research results pointed out that the expanding differences in medical services made many people call for more effective regional health coordination policies. Behera (13) explored the cyclical behavior of public health expenditure in Indian states from 2000 to 2006 based on systematic generalized moment regression, and found that public health expenditure in India was unbalanced in states, and health expenditure showed the characteristics of pro cyclical. On the basis of constructing a comprehensive evaluation index system, scientific weighting of indicators is equally important to the evaluation results of MHSS. Weighting methods such as the entropy weight method, principal component analysis, and analytic hierarchy process have been widely used in the field of comprehensive evaluation (32–34). Compared with other weighting methods, the entropy method is more widely used for some special advantages. Specifically, it can overcome the randomness and conjecture problems that cannot be avoided by the subjective weighting method and can effectively solve the problem of information overlap among multi-index variables (35).

**Figure 1:**
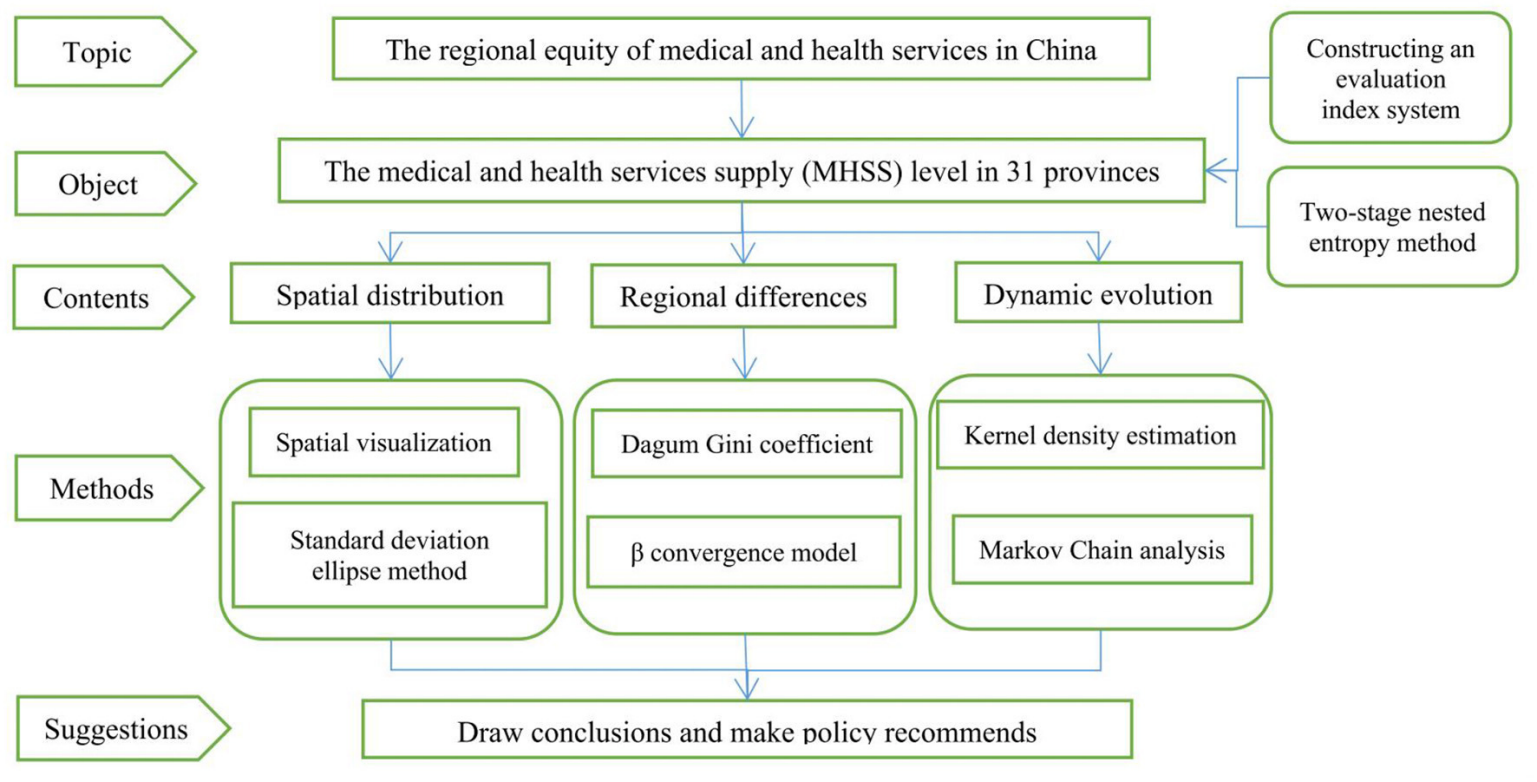
The research framework of this paper.

The existing literatures have investigated the fairness of health services from various aspects, but few studies have been carried out from the perspective of regional equity. In this paper, the in-depth analysis on the spatial-temporal characteristics of the MHSS is established. The research is mainly carried out in the following three aspects as follows. First, this paper constructs a multi-dimensional evaluation index system, providing a new feasible idea for scientifically evaluating MHSS. In terms of weight calculation, the two-stage nested entropy method (an improved entropy method) is used to weigh the indicators, so as to make the evaluation results more accurate. Second, this paper attempts to use a series of quantitative analysis tools to investigate the spatial distribution, regional differences, and dynamic evolution of the MHSS level in China. To be specific, based on the spatial visualization and standard deviation ellipse analysis, this paper approaches the spatial-temporal pattern of the MHSS in China from a quantitative view; Dagum Gini coefficient and convergence model are adopted to analyze the regional differences; Kernel density estimation and Markov chain are then used to analyze the trend of distribution and evolution. Finally, we propose some targeted policy recommendations for balancing the MHSS among regions. The findings truly and scientifically show the spatial-temporal characteristics of the MHSS in China, which provides a reference basis for effectively solving the imbalanced allocation among regions. This paper is helpful for exploring a coordinated and sustainable development path of medical and health services (Figure 1).

## Materials and methods

### Evaluation method of the MHSS level

#### The evaluation index system

Constructing an evaluation index system, a mainstream way of comprehensive evaluation, is adopted in this paper to evaluate the MHSS level. It is more scientific than the evaluation method that only selects a single input or output index, and the results are more reliable and closer to the objective reality. First, based on the characteristics of the health industry, and the principles of objectivity, comparability, representativeness and systematicness, this paper selects 18 indicators to construct an evaluation index system to evaluate the MHSS level. The indicators selected cover six dimensions, including human resources, medical and health facilities, healing ability, service utilization, primary medical and health services, and disease prevention and control. Second, the fair MHSS means that every member of society has the same rights to access to medical and health services (36). Therefore, we select a series of per capita indicators instead of total indicators in this study. Third, based on considering China's health system and the dual structure of urban and rural areas, we have added some indicators referring to primary medical care, third-class hospital and rural medical care, making the evaluation index system more consistent with Chinese characteristics. The evaluation index system is shown in Table 1 which includes indicators selection, weight distribution, unit, attribute, etc.

**Table 1:** Evaluation index system of the MHSS level.

**Target layer**	**Dimension layer**		**Indicator layer**		
	**Dimensions**	**Weight**	**Indicators**	**Units**	**Attribute**
MHSS Level	Human resources	22.41	Number of licensed or assistant doctors per thousand people	Individuals	+
			Number of registered nurses per thousand people	Individuals	+
			Number of health management personnel per thousand people	Individuals	+
			The ratio of nurses to doctors	%	-
	Medical and Health facilities	26.46	Per capita assets of medical and health institutions	Yuan	+
			Number of beds in health institutions per thousand people	Unit	+
			Number of hospitals per thousand people	Unit	+
	Healing ability	26.60	Maternal mortality rate	‰	-
			Perinatal mortality rate	‰	-
			Number of third-class hospitals per thousand people	Unit	+
	Primary medical and health services	9.56	Average number of staff in clinics in each village	Individuals	+
			Number of primary medical institutions per thousand people	Unit	+
	Service utilization	12.57	Daily visits per doctor	Times	+
			Daily inpatients per doctor	Days	+
			The bed utilization ratio	%	+
			The average length of stay	Days	-
	Disease prevention and control	2.40	Incidence rate of class A and B notifiable infectious diseases	1/100,000	-
			Death rate of class A and B notifiable infectious diseases	1/100,000	-

The weight of dimension layer is calculated by the two-stage nested entropy method.

#### The two-stage nested entropy method

Entropy method, an important objective weighting method, calculates the weight of the indicator according to the amount of information which is mainly determined by the relative change degree of index data. The greater the relative change degree, the greater the utility value of index information and the higher the information contribution to the whole system (37). However, the traditional entropy method has an application defect. In practice, to make the evaluation index system comprehensive, systematic, and organized, the “target layer”, “dimension layer” and “indicator layer” are usually included in the index system. However, the traditional entropy method crosses the “dimension layer” and only calculates and distributes weights among the “indicator layer”, which makes the existence of the “dimension layer” meaningless and affect the accuracy of the evaluation results. To make full use of the information of each layer and make the weight distribution reasonable, based on the research of Cuirong (38), the two-stage nested entropy method is used in this study to evaluate the MHSS level. The specific steps are as follows:

All original data are dimensionless to make the indicators comparable. Equations (1) and (2) are the dimensionless process of positive and negative indicators respectively:


(1) $$p^{\prime }itjk=\frac{x_{itjk}-\min\left(x_{itjk}\right)}{\max\left(x_{itjk}\right)-\min\left(x_{itjk}\right)}$$

(2) $$p^{\prime }itjk=\frac{\max\left(x_{itjk}\right)-x_{itjk}}{\max\left(x_{itjk}\right)-\min\left(x_{itjk}\right)}$$

Where *x*_*itjk*_ is the original index value of the *k* − *th* index of the dimension *j* in province *i* at period *t*.

The construction formula of a normalized matrix *p*_*itjk*_ using Equation (3):


(3) $$p_{itjk}=\frac{p^{\prime }itjk}{\sum_{t=1}^{T}\sum_{i=1}^{N}p^{\prime }itjk}$$

Calculate the entropy of the *k* − *th* indicator of the dimension j using Equation (4):


(4) $$\begin{array}{l} e_{jk}=-\frac{1}{\ln\;\left(TN\right)}\sum_{t=1}^{T}\sum_{i=1}^{N}p_{itjk}\ln\;p_{itjk} \end{array}$$

Calculate the entropy of dimension *j* using Equations (5) and (6):


(5) $$\begin{array}{l} g_{itk}=\frac{\sum_{k=1}\frac{1-e_{jk}}{\sum_{j=1}^{J}e_{jk}}p_{itjk}}{\sum_{t=1}^{T}\sum_{i=1}^{N}\sum_{k=1}^{K}\frac{1-e_{jk}}{\sum_{j}^{J}e_{jk}}p_{itjk}} \end{array}$$

(6) $$\begin{array}{l} e_{j}=-\frac{1}{\ln\;\left(TN\right)}\sum_{t=1}^{T}\sum_{i=1}^{N}g_{itk}\ln\;g_{ikt} \end{array}$$

Finally, the calculation formula of the MHSS level using Equation (7):


(7) $$\begin{array}{l} M_{it}=\sum_{j=1}^{J}\frac{1-e_{j}}{\sum_{j=1}^{J}\left(1-e_{j}\right)}g_{itk} \end{array}$$

### Methods of spatial distribution analysis

Standard deviation ellipse analysis is a spatial statistical technique to measure the distribution characteristics of geographical elements (39, 40). The distribution center of gravity, long axis standard deviation, short axis standard deviation, azimuth and other parameters can be calculated by this method. In this paper, the standard deviation ellipse analysis is used to investigate the spatial distribution characteristics of MHSS in China. The specific steps are as follows:


(8) $$\begin{array}{l} \bar{X_{w}}=\frac{\overset{n}{\underset{i=1}{\sum }}w_{i}x_{i}}{\overset{n}{\underset{i=1}{\sum }}w_{i}}\;\bar{Y_{w}}=\frac{\overset{n}{\underset{i=1}{\sum }}w_{i}y_{i}}{\overset{n}{\underset{i=1}{\sum }}w_{i}} \end{array}$$

(9) $$\begin{array}{l} \sigma_{x}=\sqrt{\frac{\overset{n}{\underset{i=1}{\sum }}{\left(w_{i}\bar{x_{i}}\cos\theta -w_{i}\bar{y_{i}}\sin\theta \right)}^{2}}{\overset{n}{\underset{i=1}{\sum }}w_{i}^{2}}}\;\\ \sigma_{y}=\sqrt{\frac{\overset{n}{\underset{i=1}{\sum }}{\left(w_{i}\bar{x_{i}}\sin\theta -w_{i}\bar{y_{i}}\cos\theta \right)}^{2}}{\overset{n}{\underset{i=1}{\sum }}w_{i}^{2}}} \end{array}$$

(10) $$\begin{array}{l} \tan\theta \\ =\frac{\left(\overset{n}{\underset{i=1}{\sum }}w_{i}^{2}\bar{x_{i}^{2}}-\overset{n}{\underset{i=1}{\sum }}w_{i}^{2}\bar{y_{i}^{2}}\right)+\sqrt{{\left(\overset{n}{\underset{i=1}{\sum }}w_{i}^{2}\bar{x_{i}^{2}}-\overset{n}{\underset{i=1}{\sum }}w_{i}^{2}\bar{y_{i}^{2}}\right)}^{2}+4\overset{n}{\underset{i=1}{\sum }}w_{i}^{2}\bar{x_{i}^{2}}\bar{y_{i}^{2}}}}{2\overset{n}{\underset{i=1}{\sum }}w_{i}^{2}\bar{x_{i}}\bar{y_{i}}} \end{array}$$

Where *w*_*i*_ represents the weight; θ represents the azimuth of the standard deviation ellipse, which is a clockwise angle formed by the true north direction and the long axis of the standard deviation ellipse; σ_*x*_ and σ_*y*_ represent the standard deviation on the x-axis and y-axis respectively.

### Methods of regional difference analysis

#### Decomposition of Dagum Gini coefficient

The Dagum Gini coefficient decomposition method is adopted to decompose the regional differences of the MHSS level in eastern, central, and western China. The total Gini coefficient G is calculated using Equations (11) and (12):


(11) $$\begin{array}{l} G=\frac{\overset{k}{\underset{j=1}{\sum }}\overset{k}{\underset{h=1}{\sum }}\overset{n_{j}}{\underset{i=1}{\sum }}\overset{n_{h}}{\underset{r=1}{\sum }}\left|y_{ji}-y_{hr}\right|}{2n^{2}ȳ} \end{array}$$

(12) $$\begin{array}{l} {Ȳ}_{h}\leq \cdots {Ȳ}_{j}\leq \cdots {Ȳ}_{k} \end{array}$$

Where *y*_*ji*_(*y*_*hr*_) is the MHSS level of provinces in region *j*(*h*); $\bar{y}$ is the average value; *n* is the number of provinces; *k* is the number of regions; *n*_*j*_(*n*_*h*_) is the number of provinces in region *j*(*h*); *G* is the overall Gini coefficient *j* and *h* are different provinces in *k* region, *j* = 1, 2, ...*k*; *i*, *r* is the different province in region *j*(*h*). When the Gini coefficient is decomposed, the average value of each region is sorted according to formula (12).

According to Dagum's Gini coefficient decomposition method (41), the overall Gini coefficient *G* is divided into three parts: the contribution of differences within regions *G*_*w*_, the contribution of difference between regions *G*_*nb*_, and the contribution of trans variation intensity *G*_*t*_. The relationship of these three parts satisfies *G* = *G*_*w*_+*G*_*nb*_ + *G*_*t*_. The calculation equations are as follows:


(13) $$\begin{array}{l} G_{w}=\overset{k}{\underset{j=1}{\sum }}G_{jj}p_{j}s_{j} \end{array}$$

(14) $$\begin{array}{l} G_{nb}=\overset{k}{\underset{j=2}{\sum }}\overset{j-1}{\underset{h=1}{\sum }}G_{jh}\left(p_{j}s_{h}+p_{h}s_{j}\right)D_{jh} \end{array}$$

(15) $$\begin{array}{l} G_{t}=\overset{k}{\underset{j=2}{\sum }}\overset{j-1}{\underset{h=1}{\sum }}G_{jh}\left(p_{j}s_{h}+p_{h}s_{j}\right)\left(1-D_{jh}\right) \end{array}$$

(16) $$\begin{array}{l} G_{jj}=\frac{\overset{n_{j}}{\underset{i=1}{\sum }}\overset{n_{j}}{\underset{r=1}{\sum }}\left|y_{ji}-y_{hr}\right|}{2n_{j}^{2}{ȳ}_{j}} \end{array}$$

(17) $$\begin{array}{l} G_{jh}=\frac{\overset{n_{j}}{\underset{i=1}{\sum }}\overset{n_{h}}{\underset{r=1}{\sum }}\left|y_{ji}-y_{hr}\right|}{n_{j}n_{h}\left({ȳ}_{j}+{ȳ}_{h}\right)} \end{array}$$

Where *G*_*jj*_ represent the Gini coefficient in region *J*; *G*_*jh*_ represents the inter-regional Gini coefficient in regions *j* and *h*. *D*_*jh*_ is the relative influence between region *i* and region *j* using Equation (18):


(18) $$\begin{array}{l} D_{jh}=\frac{d_{jh}-p_{jh}}{d_{jh}+p_{jh}} \end{array}$$

Where, the calculation formulas of *d*_*jh*_ and *p*_*jh*_ are shown in Equations (19) and (20) respectively; *d*_*jh*_ is defined as the difference of the value among regions; *p*_*jh*_ is defined as the hyper variant first moment; *F*_*h*_(*F*_*j*_) represents the cumulative density distribution function of region *j*. The Equation is:


(19) $$\begin{array}{l} d_{jh}=\int_{0}^{\infty }dF_{j}\left(y\right)\int_{0}^{y}\left(y-x\right)dF_{h}\left(x\right) \end{array}$$

(20) $$\begin{array}{l} p_{jh}=\int_{0}^{\infty }dF_{h}\left(y\right)\int_{0}^{y}\left(y-x\right)dF_{j}\left(x\right) \end{array}$$

#### β convergence model

β convergence refers to the difference between the backward regions and the developed gradually narrow, and finally reach the same level. β convergence includes absolute convergence and conditional convergence. The absolute β convergence basic model can be expressed as Equation (21):


(21) $$\begin{array}{l} \ln\;\left(\frac{y_{i,t+1}}{y_{i,t}}\right)=\alpha +\beta \ln\;y_{i,t}+\mu_{i}+\eta_{t}+\varepsilon_{it} \end{array}$$

Where ln (*y*_*i, t* + 1_/*y*_*i, t*_) represents the development rate in *j* region; β is the convergence rate; If β < 0, it means that the absolute β convergence exists, and the convergence rate is *v* = −ln (1 + β); α is a constant term; μ is the regional effect; η is the time effect; ε is a random disturbance term with independent identically distributed. Normally, conditional β convergence can make the evaluation results more convincing by adding control variables which are highly correlated with the main explanatory variable. The corresponding model is shown as Equation (22):


(22) $$\begin{array}{l} \ln\;\left(\frac{y_{i,t+1}}{y_{i,t}}\right)=\alpha +\beta \ln\;y_{i,t}+\lambda \overset{n}{\underset{j=1}{\sum }}control_{i,t}^{j}+\mu_{i}+\eta_{t}+\varepsilon_{it} \end{array}$$

Where *control* represents the control variables. The specific descriptions of control variables selected are as follows:

1. The level of economic development (PGDP). Generally, regional gross domestic product (GDP) is used to reflect the overall level of economic development in a region. The level of regional economic development plays a basic role in ensuring the supply level of basic public services. Limited to the impact of regional differences in population size, this paper uses per capita GDP as the control variable.
2. Financial self-sufficiency rate (FSS). The level of financial self-sufficiency inevitably has great impacts on the allocation of medical and health resources in a region. On the one hand, FSS cannot only reflect the number of financial resources at the disposal of local governments, but also reflect the degree of dependence of local governments on central financial transfer payments; On the other hand, it can also deeply reflect the impact of the institutional arrangement of fiscal decentralization on the financial resources and expenditure preferences of local governments. In this paper, the FSS is expressed by the ratio of the revenue within the budget to expenditure within the budget.
3. Foreign direct investment (PFDI). As an important capital factor to promote economic growth, FDI is often chased and favored by local governments at all levels in various provinces under the influence of the fiscal decentralization system with the dual attributes of political incentives and fiscal incentives. To meet the regional capital demand and promote the rapid economic growth, local government officials would make full use of their own authority to attract foreign investors to enter the area through tax relief, tax preference, financial subsidies, and other financial policy tools (42). Therefore, FDI would deepen the impact on the supply level of basic public services in a region. This paper uses the actual per capita foreign direct investment as the measurement index.
4. Urbanization rate (UR). The promotion of urbanization process is closely related to local governments. It affects all aspects of regional economy and society, which will inevitably have different driving effects on the allocation of health resources. The UR is expressed by the ratio of the urban population to the total permanent population of each province.
5. Industrial structure upgrading (ISU). In China, the development of tertiary industry is imbalanced. Promoting economic transformation to achieve the upgrading of industrial structure has become the goal of local governments in China. Local governments have taken measures to promote the in-depth expansion of potential growth fields such as service economy and knowledge economy, which will also affect the MHSS. To highlight the service-oriented feature of economic structure change and upgrading, this paper uses the ratio of the added value of the tertiary industry to the added value of the primary and secondary industries to measure the ISU (Table 2).

**Table 2:** Summary statistics of variables.

**Variables**	**Sample size**	**Mean**	**Standard deviation**	**Min**	**Max**
ln(y_i, t + 1_/y_i, t_)	465	0.0640	0.0599	−0.1327	0.5247
lny_i, t_	465	−1.7151	0.4134	−3.3242	−0.6520
lnPGDP	465	0.8888	0.5391	−0.6538	2.2936
UR	465	53.6078	14.7205	20.7143	89.5833
FSS	465	0.4958	0.2049	0.0640	0.9509
ISU	465	0.9628	0.6203	0.4243	5.1305
lnPFDI	465	2.5813	1.2929	−0.4663	5.9532

### Methods of dynamic evolution analysis

#### Kernel density estimation

Kernel density estimation, an important non-parametric estimation method, uses a smooth peak function to fit the sample data, and a continuous density curve to describe the distribution of random variables. It can reflect the distribution location, shape, and ductility of random variables (43). The density function of random variable *X* is as follows:


(23) $$\begin{array}{l} f\left(x\right)=\frac{1}{Nh}\sum_{i=1}^{N}K\left(\frac{X_{i}-x}{h}\right) \end{array}$$

Where *N* is the number of observations; *X*_*i*_ is the independent and identically distributed observations; *x* is the average value; *K* is the kernel function, and *h* is the bandwidth. As a weighting function or smooth function, the kernel function needs to meet the following conditions:


(24) $$\begin{array}{l} \{\begin{array}{l} \underset{x\to \infty }{\lim}K\left(x\right)\;x=0\\ K\left(x\right)\geq 0\;\int_{-\infty }^{+\infty }K\left(x\right)\;dx=1\\ \sup K\left(x\right)+\infty \;\int_{-\infty }^{+\infty }K^{2}\left(x\right)\;dx\;+\infty \end{array} \end{array}$$

The common kernel functions include Gaussian kernel function, triangular kernel function, and quadrangular kernel function. This paper employs the Gaussian kernel function to analyze the dynamic distribution.

#### Markov chain

Markov chain is an analytical method that reflects random processes. This paper describes the dynamic evolution characteristics of MHSS in China by constructing a state transition matrix. The Equation is:


(25) $$\begin{array}{l} p\left\{X\left(t\right)=j|X\left(t-1\right)=i,X\left(t-1\right)=i_{t-2},\ldots ,X\left(0\right)=i_{0}\right\}\\ =P\left\{X\left(t\right)=j,X\left(t-1\right)=i\right\} \end{array}$$

It follows from Equation (25) that the state of a random variable *X* in time period *T* is only affected by the pre-T period, i.e., *X* has the first-order Markov property. Moreover, the transition probability *p*_*ij*_ of a random variable *x* from state *i* to state *j* can be obtained by *n*_*ij*_/*n*_*i*_. Where, *n*_*ij*_ represents the total number of times state *i* turns into state *j* and Ni is the number of times state *i* appears. Furthermore, we can infer the evolution trend through the probability transfer matrix *P* composed of transfer probability *p*_*ij*_. The probability transition matrix *P* is as follows:


(26) $$\begin{array}{l} P=\left(\begin{array}{ccc} p_{11} & \ldots & p_{1k}\\ \vdots & \ddots & \vdots \\ p_{k1} & \cdots & p_{kk} \end{array}\right) \end{array}$$

### Data source and regional division

This paper adopts provincial data, including 496 research samples from 31 provinces in mainland China from 2005 to 2020. The data used are from the China Health Statistical Yearbook, China Health and Family Planning Statistical Yearbook, China Statistics Yearbook, and National Bureau of Statistics. In addition, the linear interpolation method is adopted to deal with the missing data. According to the 7th 5-Year Plan, China is divided into three main regions: the eastern region, the central region, and the western region (Table 3). In generally, there are significant differences in the economic development, natural resource status, and geographic climate among the three regions. Our analysis from the three regions can investigate equity of MHSS from a regional perspective.

**Table 3:** China's three main regions.

**Regions**	**Provinces**
Eastern region	Beijing, Tianjin, Hebei, Liaoning, Shanghai, Jiangsu, Zhejiang, Fujian, Shandong, Guangdong, Hainan
Central region	Shanxi, Jilin, Heilongjiang, Anhui, Jiangxi, Henan, Hubei, Hunan
Western region	Inner Mongolia, Guangxi, Chongqing, Sichuan, Guizhou, Yunnan, Tibet, Shaanxi, Gansu, Qinghai, Ningxia, Xinjiang

## Analysis on spatial distribution

### Factual description

**Figure 2:**
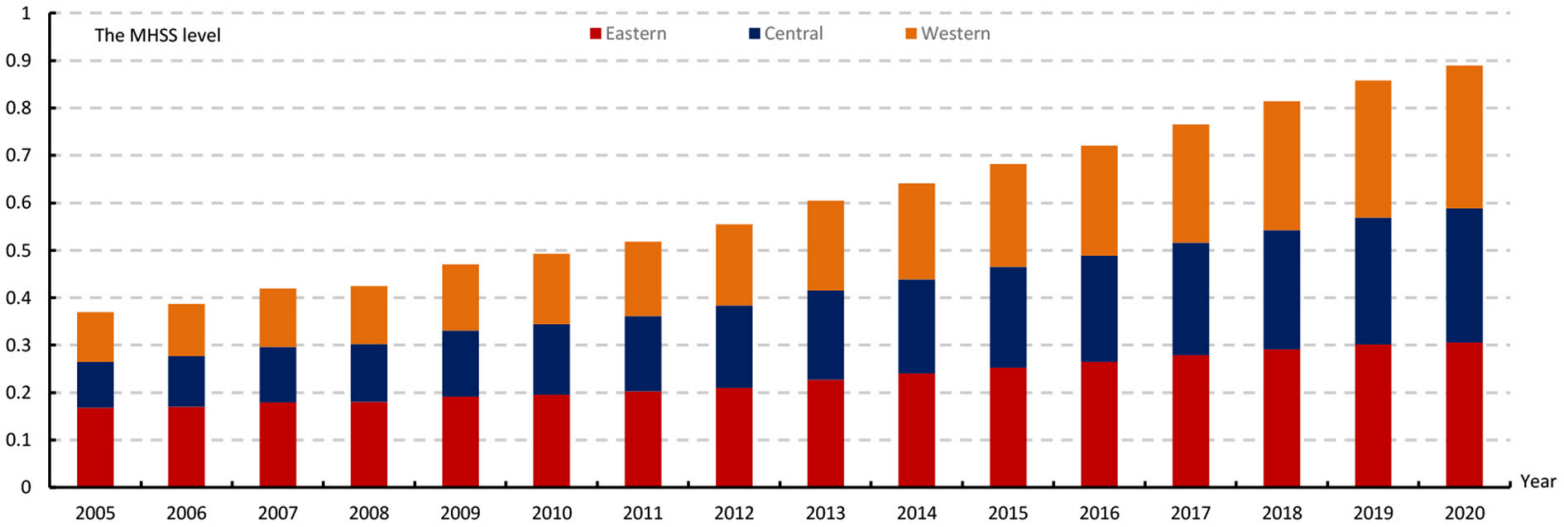
The average MHSS level of three regions from 2005 to 2020.

**Figure 3:**
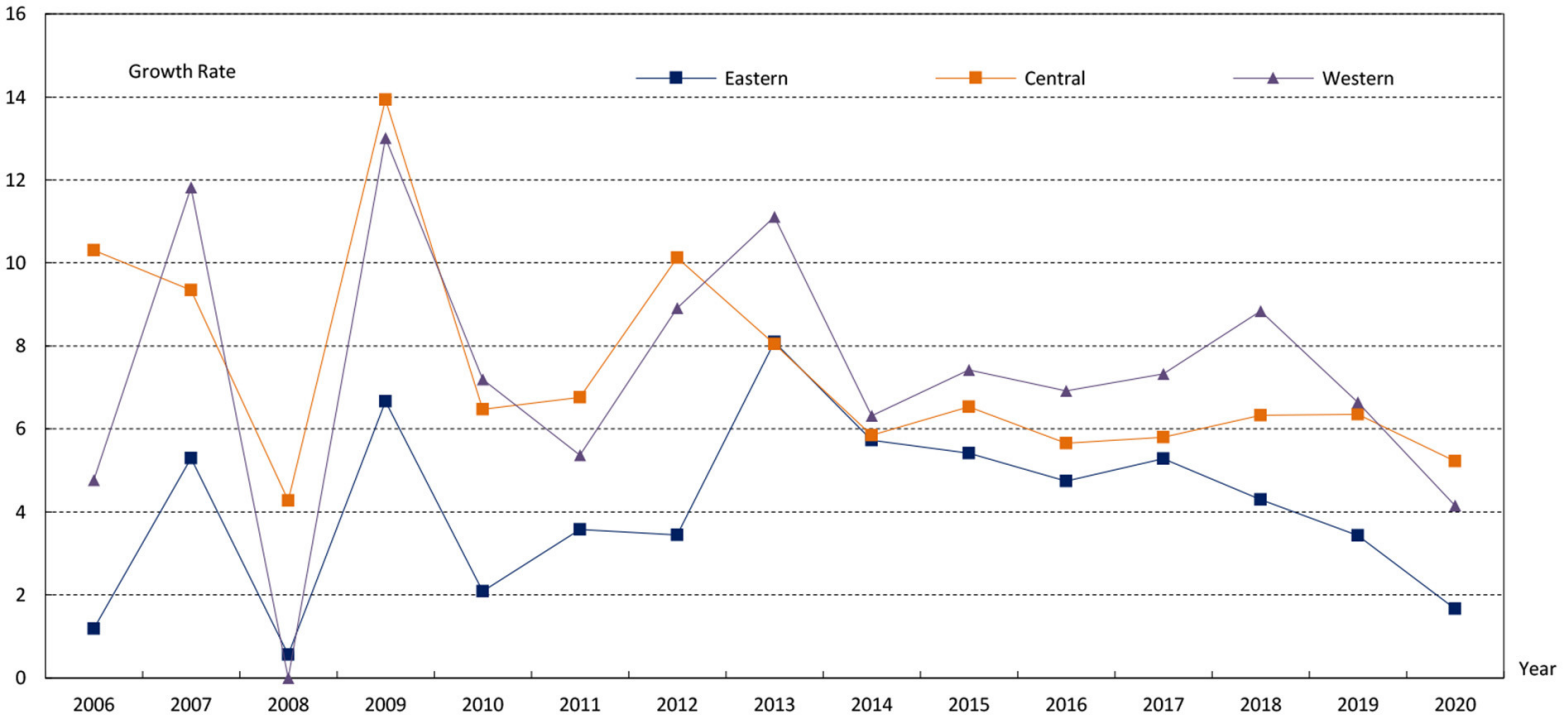
The average growth rate of three regions from 2005 to 2020.

Based on the above evaluation index system and two-stage nested entropy method, we calculated the score of 31 provinces from 2005 to 2020. The results are attached in Table A1 in Appendix. Overall, the MHSS level shows a steady and continuous upward trend, with the score rising from 0.13 in 2005 to 0.30 in 2020 and an average annual growth rate of 8.72%. From the perspective of regions (Figure 2), the MHSS level in eastern China is the highest, with the average score of 0.23. The central region ranks second with an average score of 0.18. The western region is slightly higher than the central region, with an average score of 0.19. From the growth rate (Figure 3), there are also significant differences among the three regions. Specifically, the central region has the fastest growth rate with an average annual growth rate of 12%, followed by the western region with an average annual growth rate of 11.52%. In contrast, the eastern region has the lowest growth rate, with an average annual growth rate of 5.49%.

**Figure 4:**
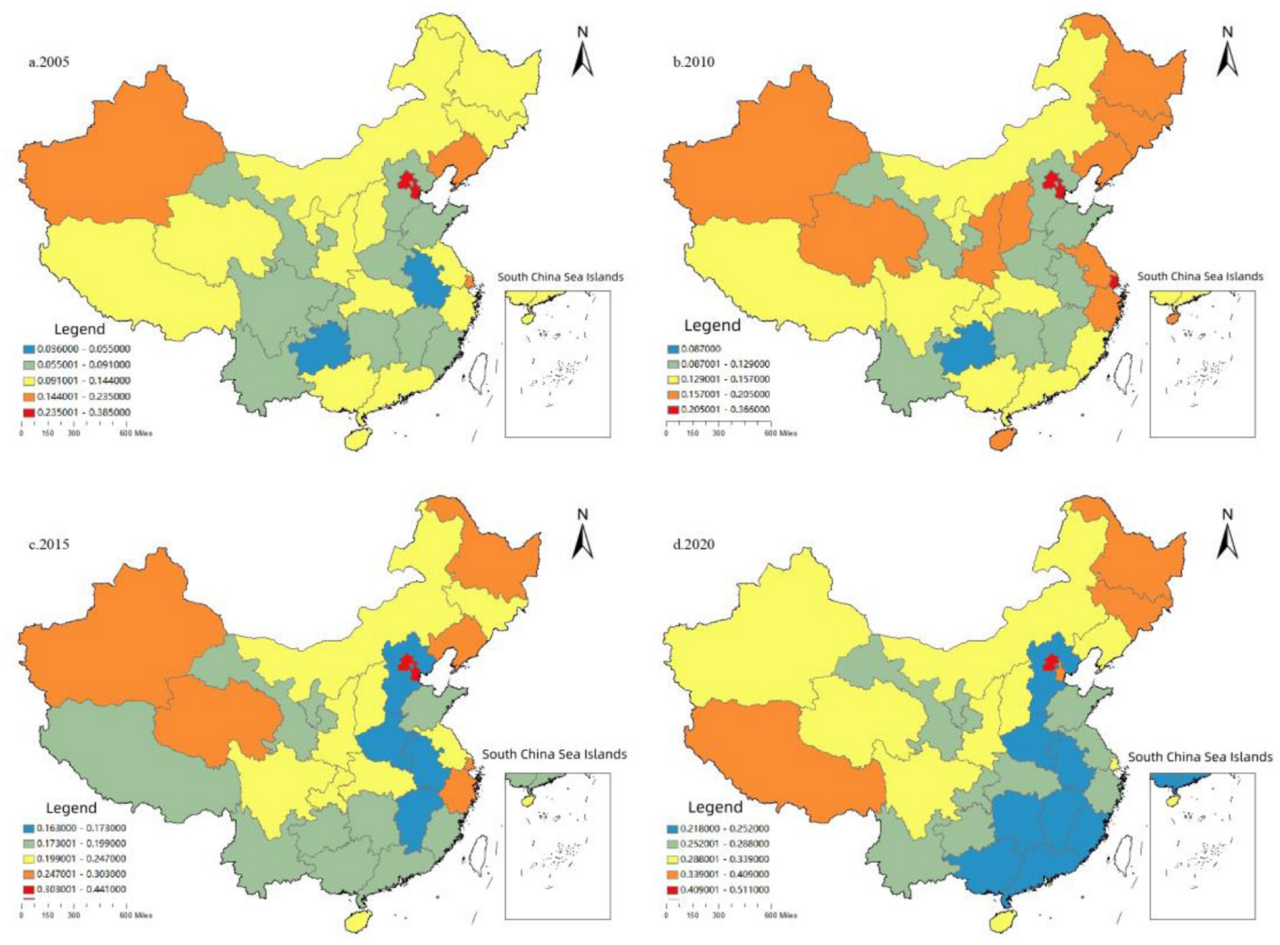
The spatial distribution of the MHSS level in China.

As shown in Figure 4, the MHSS level in various provinces shows a relatively obvious growth trend, and the differences among provinces are significantly reduced. Specifically, in 2005, the overall level in China was low with the highest level of 0.39 in Beijing and the lowest level of 0.036 in Guizhou, and there were great differences among different regions. The high level is mainly concentrated in developed eastern regions such as Beijing, Tianjin, Shanghai, and Liaoning. By comparing the spatial visualization maps of 2010, 2015, and 2020, the MHSS level has been improved, and the relative differences among provinces have been significantly reduced. For instance, only Beijing, Heilongjiang, Jilin, and Tibet were at a relatively higher level in 2020, and most regions had no obvious central point.

In addition, it is worth noting that due to the low population density and the implementation of regional development strategies such as the western region development, the MHSS level in western provinces is not as low as its economic level. For instance, in 2020, the score of Tibet was 0.409, higher than that in most eastern provinces. The reason is that Tibet's population is small, with a resident population of only 3.66 million in 2020. Therefore, the contradiction between supply and demand is easy to ease in western. However, for some economically developed provinces in other regions, the MHSS does not match their economic status. For instance, by the end of 2020, the total number of three-level hospitals in Guangdong had reached 122, ranking first in China. But from the perspective of supply and demand, the health resources per capita in Guangdong are obviously behind other provinces. Thus, it is hard for Guangdong to meet the huge demand of medical and health services. The tense contradiction between supply and demand will not only increase the difficulty and cost of seeking medical advice, but also easily lead to the risk of medical run when public health events break out such as the COVID-19.

### Analysis on spatial-temporal variations

**Figure 5:**
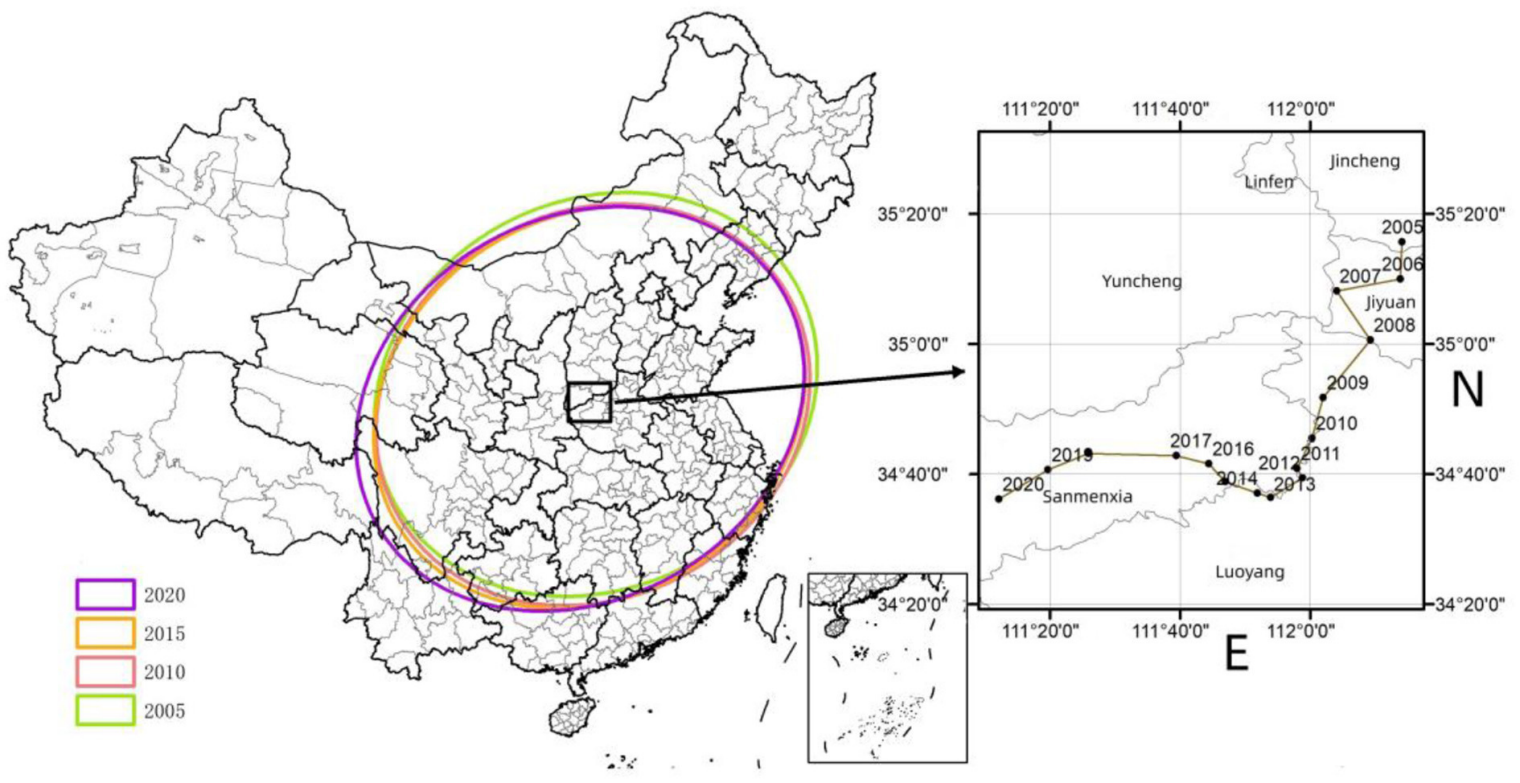
The migration trajectory of the center of the MHSS level in China.

Using the standard deviation ellipse, this paper calculated the center of gravity transfer trajectory and the specific spatial parameters from 2005 to 2020 (Figure 5). From the distribution of the center of gravity, the center of gravity moved between 111°34′-112°34′ and 34°69′-35°36′ during the sample period. Compared with China's geometric centers (103°E and 36°N), the center of gravity was in the southeast, indicating that the average MHSS level in the eastern and southern regions was higher than that in the western and northern regions. From the perspective of the trajectory and moving direction, the center of gravity showed a turning feature of “first to the south, then to the west”. In addition, the distribution center was located at the junction of Shanxi, Henan, and Shaanxi, passing through Jincheng, Jiyuan, Luoyang, and Sanmenxia in turn. In general, the center of gravity had moved the longest to the west, indicating that the level of the western region was rising faster than the national average. During the sample survey period, the standard deviation ellipse of MHSS in China was mainly located in the eastern and central regions, basically showing a “northeast- southwest” spatial distribution pattern. From the perspective of ellipse area, the ellipse area gradually decreased from 2005 to 2014, and gradually increased from 2014 to 2020, indicating that there was a trend from spatial concentration to spatial divergence in China's medical and health resources allocation. From the angle of azimuth, the change range of azimuth was small, and the change trend was relatively stable, from 62.16° in 2005 to 60.30° in 2020, indicating that the divergence direction was relatively stable. In terms of the length of the semi axle, the length of the long semi axle was shortened from 1082.05 km in 2005 to 1064.94 km in 2020, and the length of the short semi axle was increased from 1275.6 km to 1305.68 km, indicating that the MHSS in China shows a trend of centralization in the northeast and southwest, and divergence in the northwest and southeast.

## Analysis on regional differences

### Regional difference decomposition

**Figure 6:**
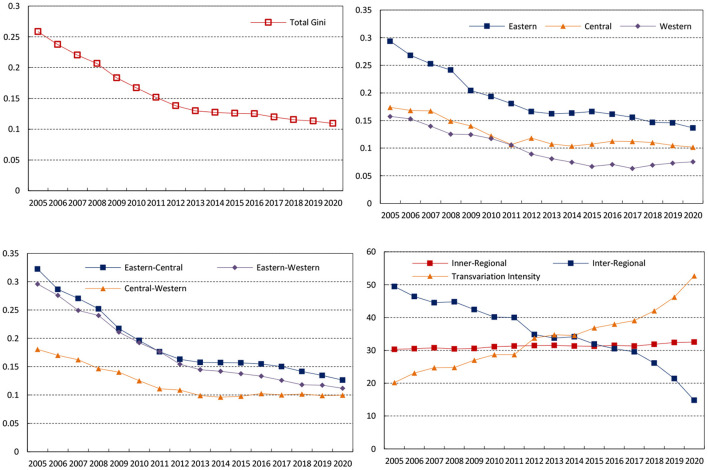
Gini coefficient variation and differential contribution rate of the MHSS level in China.

Using the Gini coefficient and decomposition method, this paper measured the overall Gini coefficient, inner-regional Gini coefficient, inter-regional Gini coefficient, and contribution rate from 2005 to 2020. The results are attached in Table A2 in Appendix. To make the results more intuitive, Figure 6 depicts the changing trend of Gini coefficient during the sample period. The overall Gini coefficient decreased from 0.2584 in 2005 to 0.1091 in 2020, with a decline rate of 57.78% and an average annual decline rate of 3.8% indicating thar the spatial distribution of health resources had become more and more reasonable.

Figure 6 depicts the changing trend of the Gini coefficient at the three regions from 2005 to 2020. The average Gini coefficients of the eastern, central, and western are 0.1900, 0.1253, and 0.0992 respectively, indicating that the inner-regional difference degree of the eastern is the largest, followed by the central and the western. Although the Gini coefficients in the three regions experienced some fluctuations, it showed a stable downward trend. Specifically, the Gini coefficient in the eastern, central, and western decreased from 0.2937, 0.1737 and 0.1576 in 2005 to 0.1368, 0.1017 and 0.0753 in 2020, with an average annual decline rate of 3.56%, 2.76% and 3.49%.

Then, the changing trend of the inter-regional difference is given in Figure 6. The average Gini coefficient between the eastern and central during the sample period was 0.1915, with the largest inter-regional difference among all regions. From the changing trend, the inter-regional differences of all the regions showed a narrowing trend, among which the decline range between the eastern and central regions was the largest, with an average annual decline rate of 4.15%. In contrast, the inter-regional difference between the central and western regions decreased only by 2.98%.

Moreover, the changing trend of the contribution rate of each decomposition term to the overall Gini coefficient from 2005 to 2020 can be seen from Figure 6. The contribution rate of the inner-regional difference and trans variation intensity increased from 30.32 and 20.21% in 2005 to 32.53 and 52.69% in 2020 respectively. The contribution rate of inter-regional difference showed a sharp decline, from 49.47% in 2005 to 14.78% in 2020. From 2005 to 2011, the inter-regional difference was the main source of the overall difference, followed by the intra-regional difference. However, with the increasing contribution rate of trans variation intensity, trans variation intensity had become the main source of overall spatial difference since 2013.

### Convergence analysis

#### Absolute β convergence analysis

Table 4 reports the absolute β convergence analysis results of the MHSS in the whole country and the three regions during the sample observation period. According to the estimation results, all the estimation coefficients are negative and have passed the significance level test of 10%, which indicates that there is absolute convergence in the whole country and the three regions. That is, in the case of similar economic development level, industrial structure, urbanization, foreign direct investment, fiscal self-sufficiency rate and other influencing factors, the development of the MHSS in various provinces will eventually converge to the same steady-state level over time. Therefore, the low-level provinces have faster growth rate than those high-level provinces, and the regional difference will gradually reduce.

**Table 4:** Results of β convergence analysis.

**Variables**	**Overall**		**Eastern**		**Central**		**Western**	
β	−0.172*** (0.022)	−0.241*** (0.028)	−0.206** (0.067)	−0.288** (0.099)	−0.267*** (0.035)	−0.321*** (0.058)	−0.185*** (0.018)	−0.216*** (0.036)
ln*PGDP*		0.078* (0.044)		0.035 (0.073)		0.135 (0.135)		0.135 (0.120)
*UR*		0.000 (0.002)		0.006 (0.005)		−0.001 (0.003)		−0.005* (0.002)
*FSS*		−0.019 (0.117)		−0.001 (0.081)		−0.475* (0.217)		0.162 (0.240)
*ISU*		−0.032 (0.039)		0.010 (0.040)		−0.003 (0.114)		−0.048 (0.114)
lnP*FDI*		0.014* (0.008)		0.011 (0.012)		0.015 (0.024)		−0.001 (0.012)
α	−0.318*** (0.048)	−0.499*** (0.153)	−0.363** (0.132)	−0.926* (0.466)	−0.540*** (0.090)	−0.454* (0.209)	−0.371*** (0.041)	−0.266 (0.168)
Regional fixed effect	yes	yes	yes	yes	yes	yes	yes	yes
Year fixed effect	yes	yes	yes	yes	yes	yes	yes	yes
*N*	465	465	165	165	120	120	180	180
Adj R^2^	0.255	0.273	0.215	0.221	0.298	0.314	0.268	0.272
*v*	0.0126	0.0184	0.0154	0.0226	0.0207	0.0258	0.0136	0.0162

Standard errors in parentheses; *p < 0.1, **p < 0.05, ***p < 0.01.

From the convergence rate of the three regions, the convergence rates of the eastern, central, and western regions are 0.0154, 0.0207 and 0.0136 respectively. The convergence rate of the central region is the fastest, followed by the eastern region and finally the western region. It is worth noting that the above conclusions are made under the assumption that the economic development level, industrial structure, urbanization rate and other factors of each province are similar. However different provinces have great heterogeneity in these factors. Ignoring these differences in empirical estimation could decrease the accuracy and reliability of the estimation results. Based on this, it is necessary to add these influencing factors as control variables for further analyze, i.e., conditional β convergence analysis.

#### Conditional β convergence analysis

The results of conditional β convergence analysis can be seen from Table 4. According to the estimation results, it is not difficult to find that all the estimation coefficients are negative and have passed the significance level test of 1%, which shows that there is conditional convergence in the whole country and the three major regions when considering other heterogeneity factors except the initial value. This also means that the MHSS is changing toward their own steady-state level. From the convergence rate, the convergence rates of the eastern, central, and western regions are 0.0226, 0.0258 and 0.0162 respectively. The results indicate that the convergence rates have also changed after considering the economic development level, financial self-sufficiency rate, urbanization rate and other factors of each province.

From the regression results of the control variables, the coefficients and significance levels of the control variables in the whole country and the three regions are different. Specifically, the regression coefficient of the level of economic development (PGDP) is only significantly positive at the national level, indicating that although the economic development can provide material support for the MHSS, it does not promote the reduction of regional disparities. However, whether the economic development can promote the convergence at the regional level remains to be further explored. The regression coefficient of urbanization rate (UR) is only significantly negative in the western region, indicating that the promotion of population urbanization helps to promote the convergence in the western region. The regression coefficient of the fiscal self-sufficiency rate (FSS) is significantly negative in the central region, which indicates that the increase of FSS has a positive effect on the reducing of regional difference. However, whether it has the same effect in other regions cannot be clearly judged. The regression coefficient of foreign direct investment (FDI) is significantly positive at the national level, indicating that the continuous inflow of foreign direct investment helps to improve the national MHSS level, but has a restraining effect on narrowing the regional gap. In addition, it is difficult to confirm whether there is a positive effect of FDI at the regional level.

## Analysis on dynamic evolution

### Kernel density estimation

**Figure 7:**
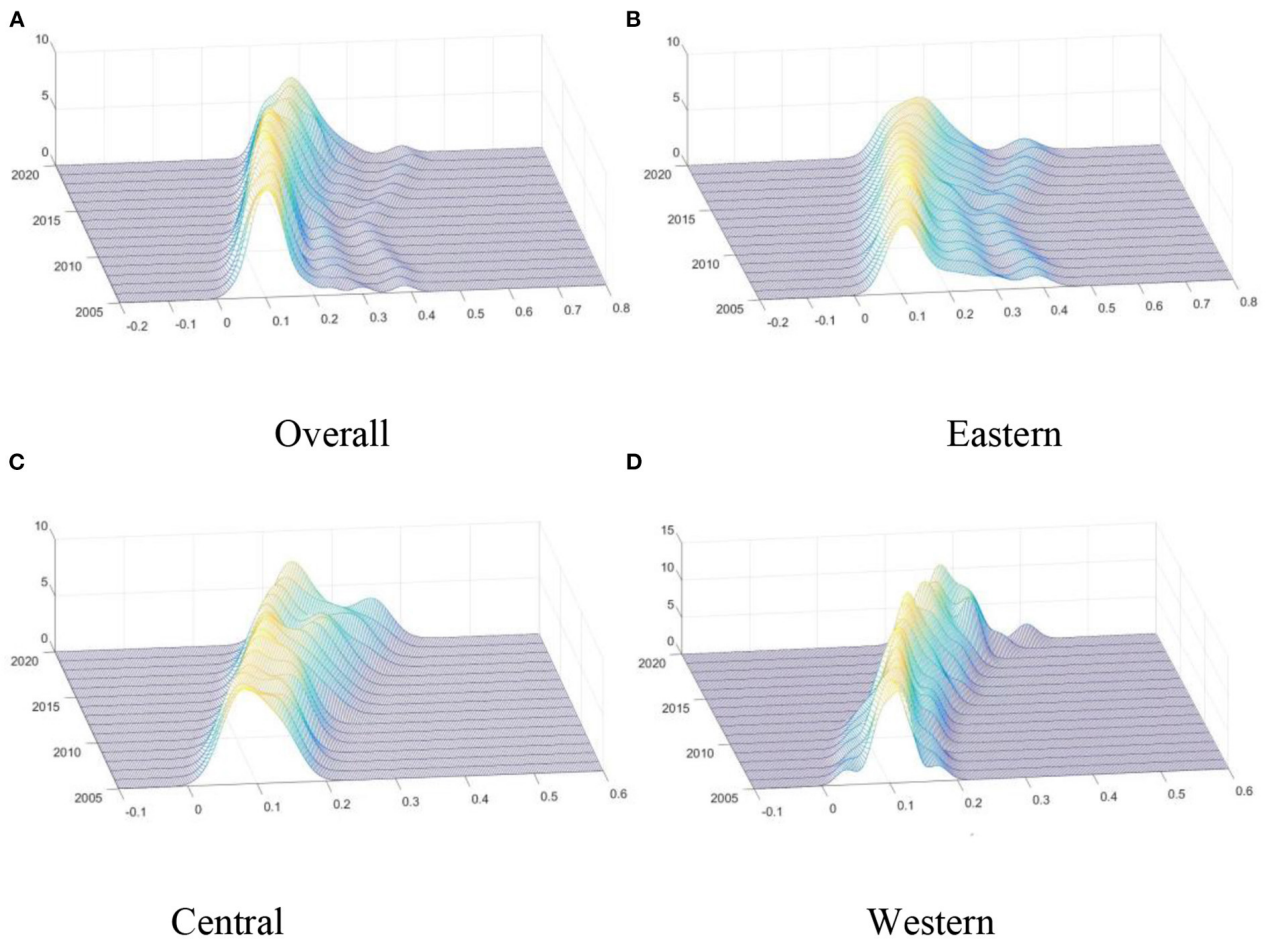
Kernel density estimation of the MHSS level. **(A)** Overall; **(B)** Eastern; **(C)** Central; **(D)** Western.

To further investigate the dynamic evolution characteristics of the supply level of medical and health services, MATLAB software was used to perform the kernel density estimation. The results are shown in Figure 7.

Figure 7A describes the dynamic evolution of the distribution of the overall MHSS level in China during the investigation period. Firstly, from the distribution position, the central point of the overall curve gradually shifted to the right side, indicating that the MHSS level was gradually increasing. Secondly, from the perspective of distribution pattern, the main peak height of the distribution curve rose slightly and then decreased sharply, and its width widened slightly, indicating that the absolute difference had an expanding trend. That is, the MHSS level among provinces were gradually dispersed, and the number of provinces deviating from the mean gradually increased. Thirdly, from the distribution ductility, the distribution curves of the whole country showed “right-dragged” phenomenon and continued to wide, indicating that the gap between high-level provinces (such as Beijing and Shanghai) and low-level provinces (such as Yunnan and Qinghai) was gradually widening. Finally, from the point of polarization, the whole has a state of “one main and one side” bimodal distribution, which can be concluded that there is a significant gradient effect in the whole country.

Then, the dynamic distribution in the eastern region during the investigation period is given in Figure 7B. Firstly, from the distribution position, the central point gradually shifted to the right side, indicating that the MHSS level in eastern region was gradually increasing. Secondly, from the perspective of distribution pattern, the main peak height of the distribution curve gradually decreased, indicating that the MHSS level in the eastern region showed a divergent trend. Thirdly, from the distribution ductility, the distribution curves showed “right-dragged” phenomenon, and the “right dragging” first shrank and then widened. Finally, from the point of polarization, the curve had a state of “one main and one side” bimodal distribution, indicating that there was a polarization phenomenon in the eastern region.

Moreover, the dynamic evolution of the distribution of the supply level of medical and health services in the central region can be seen from Figure 7C. Firstly, the central point of the curve showed a trend of moving to the right, indicating that the MHSS level in the central region was gradually improving. Secondly, the main peak height of the central distribution rose slightly and then decreased slightly. Thirdly, the curve of the central region had an initial state of “single-peak” state, and then gradually evolved into the state of “one main and one side” bimodal distribution, indicating that the phenomenon of polarization was gradually emerging.

Besides, Figure 7D describes the dynamic evolution of the distribution in the western region. Firstly, consistent with other regions, the MHSS level in the western region also showed a trend of increasing year by year. Secondly, the height of the main peak in the western region was significantly higher than that in other regions, and the width of the main peak was significantly narrower than that in other regions, indicating that the absolute difference among provinces in the western region was smaller than that in other regions. Thirdly, in most years, the curve of the western region was unimodal, indicating that there was no multipolar differentiation in the western region.

### Markov chain analysis

#### The traditional Markov chain analysis

To study the dynamic transfer trend of MHSS, the traditional Markov chain method was used in this paper. The MHSS level in 31 provinces from 2007 to 2018 was divided into four different types according to the quartile. To be specific, I represents low level (< 25%); II represents medium low level (25~50%); III represents medium high level (50~75%); IV represents high level (>75%). Based on this, the probability matrix of the state transition can be calculated, and the results are shown in Table 5.

**Table 5:** Traditional Markov probability transfer matrix of the MHSS level in China from 2005 to 2020.

	**N**	**I**	**II**	**III**	**IV**
I	122	0.7951	0.2049	0.0000	0.0000
II	124	0.0081	0.7661	0.2258	0.0000
III	118	0.0000	0.0000	0.8221	0.1779
IV	101	0.0000	0.0000	0.0000	1.0000

The diagonal elements in Table 5 represent the probability that the type has not shifted, reflecting the stability of the MHSS. The non-diagonal elements represent the probability of the transfer among different types. Based on this, the dynamic evolution characteristics of MHSS without considering spatial spillover effects are obtained. Firstly, the probability on the diagonal is always greater than that on the non-diagonal. Specifically, the probability of type I, II, III and IV maintaining the original state is 79.51, 76.61, 82.21, and 100% respectively. The probability of type I transferring to type II is 20.49%; The probability of type II to type III is 22.58%, and that of type III to type IV is 17.79%. These results show that the MHSS in China was relatively stable during the sample survey period. That is to say, the mobility between groups is small, especially the high-level provinces. Secondly, the MHSS level in China shows a significant upward trend. Specifically, the probability of upward transfer of type II and type III is 22.58 and 17.79% respectively, both higher than the probability of downward transfer. Thirdly, the transfer occurs only in the adjacent state, which indicates that the possibility of leapfrog development is very low.

#### The spatial Markov chain analysis

The traditional Markov chain analysis regards each province as an independent unit without considering the spatial spillover effects. Based on the traditional Markov probability transfer matrix, this study further constructs a spatial transfer matrix to investigate the spatial dynamic evolution from 2005 to 2020. The calculation results are shown in Table 6.

**Table 6:** Markov probability transfer matrix of the MHSS level in China from 2005 to 2020.

**Spatial Lag**	**t/t + 1**	**N**	**I**	**II**	**III**	**IV**
I	I	94	0.8617	0.1383	0.0000	0.0000
	II	27	0.0000	0.9259	0.0741	0.0000
	III	4	0.0000	0.0000	1.0000	0.0000
	IV	0	0.0000	0.0000	0.0000	0.0000
II	I	24	0.5417	0.4583	0.0000	0.0000
	II	69	0.0145	0.7971	0.1884	0.0000
	III	32	0.0000	0.0000	0.9063	0.0937
	IV	8	0.0000	0.0000	0.0000	1.0000
III	I	4	0.7500	0.2500	0.0000	0.0000
	II	25	0.0000	0.5200	0.4800	0.0000
	III	67	0.0000	0.0000	0.8209	0.1791
	IV	47	0.0000	0.0000	0.0000	1.0000
IV	I	0	0.0000	0.0000	0.0000	0.0000
	II	3	0.0000	0.6667	0.3333	0.0000
	III	13	0.0000	0.0000	0.5385	0.4615
	IV	48	0.0000	0.0000	0.0000	1.0000

Several conclusions can be derived from Table 6. Firstly, spatial factors have significant impacts on the transfer of MHSS in China. For instance, the probability of type I to type II is 20.49% without considering the factor of space. However, when considering geospatial factors, the probability of type I to type II is 13.83, 45.83, 25, and 0.0000% respectively. Secondly, except for type IV, the stability of the MHSS varies significantly according to the different types of adjacencies. For instance, the stability probability of type I in adjacent types I, II, III and IV is 86.17, 54.17, 75.00, and 0% respectively; The stability probability of type II in adjacent types I, II, III and IV is 92.59, 79.71, 52.00, and 66.67% respectively; The stability probabilities of type III in adjacent types I, II, III and IV is 100, 90.63, 82.09, and 53.85% respectively. These results indicate that the stability will decrease with the increase of the adjacency type when considering the spatial factors. Thirdly, improving the MHSS level of neighboring areas can increase the probability of upward transfer for a province. For instance, with the increase of adjacency type, the probability of upward transfer of type II is 7.41, 18.84, 48.00, and 33.33% respectively. The results indicate that the high-level provinces have a positive spatial spillover effect on adjacent regions, and there is a phenomenon of club convergence to some extent. In generally, the high-level provinces have developed economies, complete medical facilities, high-quality medical personnel, sufficient financial support. As a result, the supply structure of medical and health services in these provinces is relatively balanced, and the MHSS is maintained at a high level, forming a positive radiation, and driving effect on adjacent areas.

## Discussion

This is a comprehensive nationwide study on the spatial-temporal characteristics of the MHSS in China. Different from previous studies which focused on a certain local region (17, 18), we investigated the regional equity of MHSS across the country. In addition, different from other research that focused on a single category of health services (19, 20), this paper attempts to use a comprehensive indicator as research object, which can comprehensively and systematically measure the MHSS in China. Therefore, we have constructed a multi-dimensional comprehensive evaluation index system and used the two-stage nested entropy method to scientifically evaluate the MHSS level of 31 provinces in China. The results indicate that the accessibility and quality of medical and health services are gradually improving for residents. This upward trend is obviously related to the rapid growth of China's per capita GDP, which increased from ¥12,847 in 2005 to ¥71,828 in 2020.

However, does the improved MHSS level mean that the allocation is more equitable among regions? The answer is no, because these are two different concepts. Specifically, from the perspective of spatial distribution, we find that the MHSS level in the eastern region is the highest, followed by the western region, and the central region is the lowest. In general, China's basic public services are related to regional economic development and resource endowment, which usually shows a decreasing trend from east to west (44, 45). However, in this study, the MHSS level of the west region is higher than that of the central region. We believe that there are two main reasons for this phenomenon. First, the core idea of the evaluation index system in this paper is “every member of society has the same rights to medical and health services” (36). Thus, a series of per capita indicators were selected to evaluate the MHSS in this study. In China, the population of the central region is larger than that of the western region, which causes a relatively high MHSS level in the western region. Second, regional coordinated development strategies such as the western development have brought great positive effects on the economy and society of the western region. The basic public services, including medical and health services, have been developed rapidly in the western. Especially in 2009, the CPC Central Committee and the State Council promulgated the Opinions on Deepening the Reform of the Medical and Health System (46), in which promoting the equalization of basic public medical and health services is one of the important tasks of the 2009 medical reform policy.

The above two reasons can also explain the distribution of the center of gravity. It can be seen from Figure 5 that the center of gravity moved the largest distance west, indicating that the growth rate in the western region is higher than the national average growth rate. Moreover, as can be seen from the visualization map (Figure 4), the MHSS level in some economically developed provinces was not as high as their economic development level. These regions tend to have a large population density, which creates the severe contradiction between supply and demand of health resources. Thus, these spatial distribution characteristics also partly explains a common problem of the difficulty and high cost of getting medical service in China.

A series of quantitative analysis on the regional differences were further made in our study. The Dagum Gini coefficient show that the regional differences of the MHSS in China showed an obvious downward trend. Therefore, the conclusion of this paper is consistent with previous studies (15, 47, 48), indicating that the equalization level of medical and health services in China has been significantly improved in recent years as a whole. Specifically, the overall difference decreased significantly, with the Gini coefficient decreased from 0.2584 in 2005 to 0.1091 in 2020. The reason is that driven by the “people-oriented” development concept, the central government has successively implemented a series of regional coordinated development strategies and promoted the reform of the medical system. Moreover, with the support of policies, backward areas made full use of their late-developing advantages to increase investment in basic public services. However, while affirming this remarkable achievement, we should also realize that the Gini coefficient is still high, which is still far from the goal of “equalization”. Although this paper confirms the downward trend of overall differences, it is different from the previous literature in terms of decomposition of regional differences (15, 47, 48). From the perspective of intra-regional differences, the imbalance degree of the eastern region is the largest, followed by the central region, and the central region is the lowest. Even in the same region, different provinces have obvious differences in location, resource endowment, economic scale, financial capacity. For instance, in the eastern region Beijing is adjacent to Hebei and Guangdong is adjacent to Hainan. In the western region, Sichuan is adjacent to Tibet and Shaanxi is adjacent to Xinjiang. There are many differences among these provinces. In contrast, there are relatively few provinces in the central region. Except Jilin and Heilongjiang which are concentrated in the northeast, the rest of the provinces of the central region are relatively concentrated and their development is similar in various aspects. Thus, the MHSS is more balanced in the central region. From the inter-regional differences, the imbalance between eastern and central is the highest, and the imbalance between central and western is the lowest. Whether in terms of economic development, financial capacity, infrastructure, and industrial layout, or in the construction of medical and health services system, the eastern region is significantly better than the central and western regions. As a comparison, the central and western regions are generally backward in economic, social development and public service construction, so the relative gap between central and western is small (44, 45). In addition, trans variation intensity is used to measure the degree of cross overlap among regions. A high value means that there are low-level provinces in high-level regions, such as Hebei and Fujian in the eastern region. The MHSS level of these provinces is lower than the high-level provinces in low-level regions, such as Hubei in the central region and Tibet in the western region.

We also used the β convergence analysis to predict the trend of regional differences. The results suggest that the regional differences will narrow, and the MHSS level of different provinces will reach the convergence state. This conclusion is consistent with the research in recent years (47), but this paper further explores the impact of different factors on the regional equity. It is worth noting that the influencing factors of convergence vary in different regions, which indicates that different regions should formulate differentiated policies based on their own development characteristics. Specifically, the provinces in the central should improve the financial self-sufficiency rate, and the western region should improve the urbanization rate.

Finally, the kernel density estimation and Markov chain were used to analyze the dynamic evolution of MHSS. The results of kernel density estimation indicate that although the MHSS level in China has been significantly improved, the gap between high-level provinces and low-level provinces still exists, and it is difficult to achieve equilibrium in the short term. As medical and health services are an important part of basic public services, this distribution feature is similar to the distribution of residents' health status in China (49). Especially in the eastern and central regions, there were obvious gradient effects during the sample period. The results of Markov chain analysis show that the distribution state of the MHSS is relatively stable in China. During the sample period, the MHSS level in China showed an upward trend, which means that the low-level medical and health services will disappear and gradually tend to a high level. The spatial Markov chain shows that high-level provinces have positive spatial spillover effects, indicating the radiation effects of high-level regions should be emphasized. Referring to the relevant research of spatial analysis (50, 51), this paper shows that medical service supply has spatial spillover effect through spatial Markov chain analysis. Thus, the developed regions and backward regions should be guided by the “sharing” concept, fully using modern information technology to build an interaction platform, to realize the collaborative communication in medical equipment, technology, staff, and outcomes (52). In addition, it needs to be noted that there is a certain probability of negative shift. Thus, the governments should pay attention to the degradation problem when the MHSS is at a lower level.

Although the current research has confirmed the potential contribution to the evaluation of MHSS in China, there are still some limitations that need to be studied and solved in the future. Firstly, due to the lack of data from Taiwan Province, Hong Kong and Macao, this paper only covers the data of 31 provinces, resulting in incomplete evaluation of the MHSS in China. More extensive data collection is needed in future studies to conduct a comprehensive investigation. Secondly, only the factors that directly affect MHSS are discussed econometrically, and the current research lacks an evaluation of a broader scope or a more comprehensive evaluation. Thus, the scope of the survey should be expanded in the future study to be more generalizable. Thirdly, the subjective weighting method should be appropriately combined to determine the weight of evaluation indicators more scientifically in the future study. Finally, the influence of some factors on the convergence of the MHSS is still uncertain, including economic growth urbanization and financial self-sufficiency. To explore the impact of these uncertain factors, future research needs to carry out detailed empirical analysis, including instrumental variable method and robustness analysis.

## Conclusions and policy recommendations

Based on the provincial panel data of medical and health services from 2005 to 2020, this paper constructed an evaluation index system and used the two-stage nested entropy method to measure the MHSS level in various provinces in China. On this basis, standard deviation ellipse was used to investigate the spatial distribution, and then the Dagum Gini coefficients and β convergence methods were used to analyze the regional differences. To accurately describe and predict the evolution trend, we further used kernel density estimation and Markov chain to investigate the dynamic evolution. The main conclusions of this study are as follows:

First, from the spatial distribution, during the investigation period, the MHSS level in China had been significantly improved. However, there are obvious spatial differences among provinces, and the growth rate of each region is different. Specifically, the MHSS level in the eastern is the highest, followed by the western and the central. However, as the growth rate of the central is higher than that of the western and central in turn, the gap between the three major regions is gradually narrowing. From the spatial-temporal variations, the MHSS in China showed a trend of centralization in the northeast and southwest, and spatial divergence in the northwest and southeast, while the center of gravity moved to the southeast.

Second, from the regional differences, the overall, intra-regional, and inter-regional Gini coefficient showed a downward trend during the observation period, indicating that the imbalance problem of MHSS had been gradually alleviated in China. The intra-difference is greatest in the eastern region, followed by the central. The difference of east-central is the biggest, followed by the difference of east-west and central-west. In terms of the sources of the overall difference, the main source of the overall difference had shifted from inter-regional differences to the trans variation intensity since 2013. In terms of the evolutionary trend of regional differences, both the absolute convergence test and the conditional convergence test indicate that the MHSS in the whole country and the three major regions show a convergence trend. In addition, the economic development, urbanization rate, fiscal self-sufficiency rate, and foreign direct investment had significant impacts on the convergence.

Third, from the dynamic evolution, kernel density estimation indicates that the MHSS level in the whole country and three major regions was on the rise; The eastern and central regions had polarization and gradient effect, while the balance of the MHSS level in the western region has improved. From the dynamic characteristics of the Markov chain analysis, there was an upward transfer of MHSS in China during the observation period, and the probability of leapfrog transfer occurring was small; The spatial factor had a significant effect on the dynamic transfer; The high-level provinces had positive spatial spillover effects.

Based on the above conclusions, the following specific policy recommendations are formulated as follows:

1. The Governments Should pay Full Attention to the Significant Spatial Imbalance of MHSS in China. On This Basis, the Deep-Seated Reasons and Driving Factors That Hinder the Regional Equity Should be Explored, Such as the Level of Economic Development, Industrial Structure Upgrading, Urbanization Process, Financial Self-Sufficiency Rate, Foreign Direct Investment and Other key Factors Involved in This Paper. Then, to Speed up the Narrowing of the gap Between Regions, all Provinces Need to Actively Take Measures to Remove Obstacles and Strengthen Their own Advantages to Promote the Improvement of Their own Medical and Health Services. In Addition, Considering the Special Circumstances, Local Governments Need to Formulate Targeted Regional Oriented Policies According to Local Conditions.
2. The Central Government Should Deepen the Structural Reform of the Supply Side of Medical and Health Services and Form an Efficient, Coordinated and Sustainable Medical and Health Service Supply System; Local Governments Should Strengthen the Training of Medical Personnel, and Formulate Talent Support Policies to Encourage Medical and Health Personnel to Flow to Relatively Backward Areas; The Hierarchical Management System of Medical and Health Services Should be Improved, so as to Improve the Service Capacity of Grassroots Institutions as Well as the Convenience and Accessibility of Medical and Health Services.
3. According to the Regional Differences in the Demand for Medical and Health Services, the Governments Should Establish Institutions to Coordinate the Supply and Demand of Medical and Health Services, so That We can Understand the Dynamics of Supply and Demand in Various Regions and Make Coordinate Policies. Especially in the Economically Developed Eastern Regions, the Investment in Health Resources Should Match the Size of the Population, to Improve the Regional Adaptability, Accessibility, and Pertinence of Medical and Health Services.
4. The Governments Should Strengthen the Interaction Among Provinces and Practice the Concept of Shared Development. Because Trans Variation Intensity Is the Main Source of the Overall Difference, all Regions Should Strengthen Their Internal Cooperation to Promote the Sharing of Medical and Health Services and Improve the Efficiency of Resources Allocation. Inter-Provincial Interest Barriers and Policy Barriers Should be Broken Down, and the Developed Provinces Should Play a Positive Role in Driving the Development of Backward Provinces. In Addition, Backward Provinces Should Actively Introduce Advanced Technology and Improve the Training Mechanism of Medical Talents and Innovation Ability, to Avoid Long-Term Dependence on the Support of Developed Provinces.
5. The three Regions Should Formulate Their own Development Strategies. Specifically, Although the MHSS Level in the Eastern Region Is the Highest, the Problem of Large Internal Differences Is Serious. Measures Should be Taken to Balance the gap Among Eastern Provinces. For the Central Region, Although the gap Among Provinces Is Small, the MHSS Level Is low due to the Dense Population. Therefore, Attention Should be Paid to the Improvement of the Overall Supply Level. Due to the Small Population Size, the MHSS Level of the Western Region Is High. To Maintain Its High Supply Level, the Western Region Needs to Strengthen Its Connection With the Eastern Region and Introduce Advanced Medical and Health Services.

## Data availability statement

The original contributions presented in the study are included in the article/Supplementary material, further inquiries can be directed to the corresponding author.

## Author contributions

BC: conceptualization, data analyses, and writing of the manuscript. FJ: critical review of the manuscript. All authors contributed to the article and approved the submitted version.

## Funding

This research was funded by the Key Project of National Social Science Foundation of China (Grant No. 18ZDA077).

## Conflict of interest

The authors declare that the research was conducted in the absence of any commercial or financial relationships that could be construed as a potential conflict of interest.

## Publisher's note

All claims expressed in this article are solely those of the authors and do not necessarily represent those of their affiliated organizations, or those of the publisher, the editors and the reviewers. Any product that may be evaluated in this article, or claim that may be made by its manufacturer, is not guaranteed or endorsed by the publisher.
