# Supplementary material for: Spatial distribution, regional differences, and dynamic evolution of the medical and health services supply in China
Source: Front Public Health. 2022 Sep 23;10:1020402. doi: 10.3389/fpubh.2022.1020402 (PMC9540227; doi:10.3389/fpubh.2022.1020402)
Supplement: Supplementary file 1 [file Data_Sheet_1.docx]

**Appendix A**

**Table A.1**

The medical and health services (MHSS) level from 2005- 2020

| Regions | Provinces | The medical and health services (MHSS) level | | | | | | | | | | | | | | | |
| --- | --- | --- | --- | --- | --- | --- | --- | --- | --- | --- | --- | --- | --- | --- | --- | --- | --- |
|  |  | 2005 | 2006 | 2007 | 2008 | 2009 | 2010 | 2011 | 2012 | 2013 | 2014 | 2015 | 2016 | 2017 | 2018 | 2019 | 2020 |
| Eastern | Beijing | 0.385 | 0.382 | 0.393 | 0.378 | 0.373 | 0.366 | 0.365 | 0.361 | 0.392 | 0.404 | 0.441 | 0.458 | 0.481 | 0.492 | 0.521 | 0.511 |
|  | Tianjin | 0.298 | 0.271 | 0.27 | 0.278 | 0.275 | 0.276 | 0.28 | 0.28 | 0.287 | 0.332 | 0.347 | 0.357 | 0.371 | 0.377 | 0.388 | 0.379 |
|  | Hebei | 0.086 | 0.088 | 0.106 | 0.102 | 0.111 | 0.118 | 0.126 | 0.131 | 0.143 | 0.151 | 0.163 | 0.177 | 0.197 | 0.215 | 0.221 | 0.232 |
|  | Liaoning | 0.189 | 0.186 | 0.193 | 0.196 | 0.201 | 0.205 | 0.208 | 0.221 | 0.245 | 0.252 | 0.261 | 0.281 | 0.294 | 0.309 | 0.310 | 0.309 |
|  | Shanghai | 0.235 | 0.229 | 0.246 | 0.244 | 0.253 | 0.261 | 0.262 | 0.26 | 0.284 | 0.295 | 0.303 | 0.313 | 0.323 | 0.335 | 0.340 | 0.339 |
|  | Jiangsu | 0.112 | 0.132 | 0.137 | 0.141 | 0.157 | 0.171 | 0.180 | 0.187 | 0.203 | 0.215 | 0.223 | 0.235 | 0.243 | 0.260 | 0.271 | 0.28 |
|  | Zhejiang | 0.117 | 0.134 | 0.148 | 0.153 | 0.163 | 0.172 | 0.194 | 0.205 | 0.222 | 0.238 | 0.256 | 0.266 | 0.278 | 0.279 | 0.286 | 0.288 |
|  | Guangdong | 0.117 | 0.128 | 0.142 | 0.143 | 0.148 | 0.154 | 0.162 | 0.167 | 0.175 | 0.179 | 0.186 | 0.196 | 0.202 | 0.211 | 0.218 | 0.218 |
|  | Hainan | 0.144 | 0.143 | 0.145 | 0.147 | 0.155 | 0.168 | 0.169 | 0.193 | 0.205 | 0.209 | 0.218 | 0.231 | 0.243 | 0.268 | 0.277 | 0.317 |
|  | Fujian | 0.085 | 0.087 | 0.092 | 0.100 | 0.169 | 0.148 | 0.155 | 0.160 | 0.170 | 0.183 | 0.187 | 0.191 | 0.196 | 0.206 | 0.218 | 0.225 |
|  | Shandong | 0.083 | 0.089 | 0.096 | 0.103 | 0.112 | 0.122 | 0.133 | 0.146 | 0.167 | 0.186 | 0.199 | 0.212 | 0.238 | 0.253 | 0.265 | 0.267 |
|  | Average | 0.168 | 0.170 | 0.179 | 0.180 | 0.192 | 0.196 | 0.203 | 0.210 | 0.227 | 0.240 | 0.253 | 0.265 | 0.279 | 0.291 | 0.301 | 0.306 |
| Central | Shanxi | 0.105 | 0.131 | 0.14 | 0.148 | 0.187 | 0.185 | 0.189 | 0.205 | 0.219 | 0.227 | 0.239 | 0.261 | 0.271 | 0.285 | 0.294 | 0.31 |
|  | Jilin | 0.143 | 0.155 | 0.167 | 0.165 | 0.172 | 0.178 | 0.185 | 0.201 | 0.214 | 0.225 | 0.247 | 0.265 | 0.277 | 0.314 | 0.322 | 0.355 |
|  | Heilongjiang | 0.139 | 0.141 | 0.164 | 0.166 | 0.18 | 0.194 | 0.205 | 0.245 | 0.257 | 0.269 | 0.283 | 0.298 | 0.326 | 0.334 | 0.359 | 0.366 |
|  | Anhui | 0.055 | 0.066 | 0.075 | 0.086 | 0.098 | 0.112 | 0.129 | 0.141 | 0.151 | 0.167 | 0.173 | 0.178 | 0.191 | 0.203 | 0.219 | 0.241 |
|  | Jiangxi | 0.073 | 0.073 | 0.081 | 0.085 | 0.115 | 0.129 | 0.143 | 0.154 | 0.163 | 0.164 | 0.17 | 0.176 | 0.192 | 0.202 | 0.222 | 0.230 |
|  | Henan | 0.072 | 0.08 | 0.083 | 0.089 | 0.096 | 0.108 | 0.121 | 0.135 | 0.151 | 0.156 | 0.165 | 0.174 | 0.184 | 0.200 | 0.210 | 0.226 |
|  | Hubei | 0.107 | 0.118 | 0.128 | 0.134 | 0.146 | 0.154 | 0.163 | 0.173 | 0.19 | 0.213 | 0.236 | 0.242 | 0.25 | 0.258 | 0.27 | 0.274 |
|  | Hunan | 0.081 | 0.088 | 0.101 | 0.106 | 0.118 | 0.123 | 0.129 | 0.136 | 0.155 | 0.171 | 0.185 | 0.194 | 0.206 | 0.223 | 0.244 | 0.252 |
|  | Average | 0.097 | 0.107 | 0.117 | 0.122 | 0.139 | 0.148 | 0.158 | 0.174 | 0.188 | 0.199 | 0.212 | 0.224 | 0.237 | 0.252 | 0.268 | 0.282 |
| Western | Inner -Mongolia | 0.114 | 0.118 | 0.137 | 0.129 | 0.153 | 0.157 | 0.161 | 0.179 | 0.203 | 0.213 | 0.233 | 0.254 | 0.274 | 0.309 | 0.329 | 0.338 |
|  | Guangxi | 0.105 | 0.105 | 0.116 | 0.121 | 0.129 | 0.146 | 0.157 | 0.165 | 0.181 | 0.186 | 0.189 | 0.196 | 0.203 | 0.21 | 0.224 | 0.233 |
|  | Chongqing | 0.091 | 0.106 | 0.113 | 0.115 | 0.127 | 0.145 | 0.15 | 0.164 | 0.185 | 0.193 | 0.221 | 0.238 | 0.249 | 0.265 | 0.28 | 0.284 |
|  | Sichuan | 0.089 | 0.092 | 0.102 | 0.106 | 0.122 | 0.139 | 0.155 | 0.178 | 0.194 | 0.21 | 0.217 | 0.23 | 0.244 | 0.26 | 0.284 | 0.299 |
|  | Guizhou | 0.036 | 0.046 | 0.062 | 0.063 | 0.075 | 0.087 | 0.100 | 0.144 | 0.173 | 0.184 | 0.199 | 0.212 | 0.226 | 0.236 | 0.254 | 0.265 |
|  | Yunnan | 0.081 | 0.081 | 0.091 | 0.096 | 0.099 | 0.113 | 0.12 | 0.131 | 0.152 | 0.165 | 0.188 | 0.205 | 0.231 | 0.248 | 0.266 | 0.283 |
|  | Tibet | 0.123 | 0.111 | 0.142 | 0.145 | 0.151 | 0.143 | 0.153 | 0.139 | 0.16 | 0.173 | 0.196 | 0.199 | 0.233 | 0.315 | 0.362 | 0.409 |
|  | Shaanxi | 0.123 | 0.128 | 0.131 | 0.140 | 0.152 | 0.178 | 0.184 | 0.197 | 0.218 | 0.228 | 0.238 | 0.251 | 0.264 | 0.28 | 0.305 | 0.306 |
|  | Gansu | 0.086 | 0.093 | 0.105 | 0.103 | 0.13 | 0.122 | 0.132 | 0.157 | 0.162 | 0.190 | 0.196 | 0.204 | 0.222 | 0.239 | 0.255 | 0.275 |
|  | Qinghai | 0.122 | 0.148 | 0.165 | 0.146 | 0.191 | 0.198 | 0.208 | 0.223 | 0.245 | 0.266 | 0.271 | 0.285 | 0.305 | 0.313 | 0.320 | 0.324 |
|  | Ningxia | 0.121 | 0.121 | 0.132 | 0.14 | 0.15 | 0.156 | 0.162 | 0.164 | 0.179 | 0.185 | 0.198 | 0.233 | 0.249 | 0.265 | 0.265 | 0.275 |
|  | Xinjiang | 0.171 | 0.172 | 0.18 | 0.175 | 0.187 | 0.198 | 0.204 | 0.213 | 0.227 | 0.233 | 0.257 | 0.279 | 0.288 | 0.31 | 0.318 | 0.323 |
|  | Average | 0.105 | 0.110 | 0.123 | 0.123 | 0.139 | 0.149 | 0.157 | 0.171 | 0.190 | 0.202 | 0.217 | 0.232 | 0.249 | 0.271 | 0.289 | 0.301 |

**Table A.2**

Gini coefficient and decomposition from 2005 to 2020

| Year | Overall | Intra-Regional | | | Inter-Regional | | | Contribution Rate（%） | | |
| --- | --- | --- | --- | --- | --- | --- | --- | --- | --- | --- |
|  |  | Eastern | Central | Western | Eastern-Central | Eastern-Western | Central-Western | Intra-  Regional | Inter-  Regional | Intensity of Transvaration |
| 2005 | 0.2584 | 0.2937 | 0.1737 | 0.1576 | 0.3224 | 0.2958 | 0.1806 | 30.32 | 49.47 | 20.21 |
| 2006 | 0.2376 | 0.2679 | 0.1681 | 0.1530 | 0.2865 | 0.2758 | 0.1701 | 30.49 | 46.41 | 23.10 |
| 2007 | 0.2203 | 0.2530 | 0.1673 | 0.1398 | 0.2704 | 0.2492 | 0.1622 | 30.77 | 44.55 | 24.69 |
| 2008 | 0.2067 | 0.2415 | 0.1490 | 0.1256 | 0.2519 | 0.2402 | 0.1468 | 30.45 | 44.76 | 24.79 |
| 2009 | 0.1833 | 0.2045 | 0.1401 | 0.1248 | 0.2173 | 0.2110 | 0.1405 | 30.57 | 42.41 | 27.01 |
| 2010 | 0.1671 | 0.1936 | 0.1223 | 0.1173 | 0.1964 | 0.1925 | 0.1253 | 31.14 | 40.17 | 28.69 |
| 2011 | 0.1515 | 0.1808 | 0.1064 | 0.1058 | 0.1764 | 0.1766 | 0.1111 | 31.30 | 40.00 | 28.70 |
| 2012 | 0.1381 | 0.1662 | 0.1182 | 0.0893 | 0.1631 | 0.1546 | 0.1088 | 31.42 | 34.85 | 33.73 |
| 2013 | 0.1299 | 0.1623 | 0.1072 | 0.0811 | 0.1577 | 0.1447 | 0.0992 | 31.50 | 33.75 | 34.75 |
| 2014 | 0.1274 | 0.1633 | 0.1038 | 0.0747 | 0.1570 | 0.1421 | 0.0961 | 31.32 | 34.14 | 34.53 |
| 2015 | 0.1258 | 0.1661 | 0.1075 | 0.0669 | 0.1569 | 0.1377 | 0.0978 | 31.26 | 31.91 | 36.83 |
| 2016 | 0.1251 | 0.1616 | 0.1126 | 0.0706 | 0.1550 | 0.1333 | 0.1025 | 31.51 | 30.49 | 38.00 |
| 2017 | 0.1196 | 0.1560 | 0.1123 | 0.0633 | 0.1500 | 0.1259 | 0.1000 | 31.33 | 29.59 | 39.07 |
| 2018 | 0.1154 | 0.1466 | 0.1101 | 0.0695 | 0.1415 | 0.1182 | 0.1016 | 31.87 | 26.11 | 42.02 |
| 2019 | 0.1133 | 0.1457 | 0.1051 | 0.0730 | 0.1347 | 0.1174 | 0.0990 | 32.41 | 21.38 | 46.22 |
| 2020 | 0.1091 | 0.1368 | 0.1017 | 0.0752 | 0.1262 | 0.1119 | 0.0997 | 32.53 | 14.78 | 52.69 |
| Average | 0.1580 | 0.1900 | 0.1253 | 0.0992 | 0.1915 | 0.1767 | 0.1213 | 31.26 | 35.30 | 33.44 |
